# Proximal Splenorenal Shunt in Non-cirrhotic Portal Hypertension: Our Experience

**DOI:** 10.7759/cureus.116424

**Published:** 2026-09-17

**Authors:** Tarun Kalra, Vikas Sachdeva

**Affiliations:** 1 Gastrointestinal Surgery, Motilal Nehru Medical College, Prayagraj, IND; 2 Cardiothoracic Surgery, Motilal Nehru Medical College, Prayagraj, IND

**Keywords:** ehpvo, lienorenal shunt, ncpf, non-cirrhotic portal hypertension, portal hypertension, proximal splenorenal shunt, psrs, splenectomy, variceal bleed

## Abstract

Background

Non-cirrhotic portal hypertension (NCPH) is characterized by an elevated portal pressure without liver disease and without obvious venous thrombosis. It is characterized by splenomegaly and gastroesophageal varices with intact liver histology. Extrahepatic portal vein obstruction (EHPVO) and non-cirrhotic portal fibrosis (NCPF) come under the umbrella of NCPH. EHPVO is further characterized by portal cavernoma, cholangiopathy, and growth retardation. Surgical shunts remain a definitive option for patients requiring frequent endoscopies. This study was done to evaluate the surgical outcomes of proximal splenorenal shunt (PSRS) in patients of NCPH.

Methods

Ten NCPH patients underwent PSRS at Moti Lal Nehru Medical College, Prayagraj, between April 2024 and April 2026. Preoperative workup included routine blood tests, Doppler of the splenoportal axis, and contrast-enhanced computed tomography (CECT) of the abdomen for anatomical delineation. Preoperative triple vaccination was done in all the patients. A reverse Makucchi incision was used. All the patients were put on anticoagulant and underwent a follow-up Doppler examination. Perioperative events, complications, and short-term outcomes were recorded.

Results

All the patients had grade II-V splenomegaly, cytopenias, and preserved liver function. The mean duration of surgery was 295 minutes, and the blood loss was 490 ml. Six patients made an uneventful recovery. Two patients developed post-operative bleeding due to anticoagulant overdose and one of them required re-exploration. One patient developed hemothorax after central venous line insertion, which was managed with thoracocentesis.

Conclusion

PSRS offers an effective, one-time treatment for NCPH with manageable complications and favorable outcomes.

## Introduction

Non-cirrhotic portal hypertension (NCPH) is a term used for a group of diseases that are characterized by portal hypertension with a preserved hepatic architecture [1]. It includes both non-cirrhotic portal fibrosis (NCPF) and extrahepatic portal vein obstruction (EHPVO). Patients commonly present with splenomegaly, hypersplenism, and variceal bleeding.

Endoscopic therapy remains first-line management for variceal bleeding. In non-responsive, recurrent or refractory cases, surgical shunts like the proximal splenorenal shunt (PSRS), first described by Linton in 1947, offers durable decompression of the portal system [2] and addresses both bleeding and cytopenias.

This case series documents 10 patients with NCPH who were managed surgically by PSRS, highlighting peri-operative outcomes and complications.

## Materials and methods

This is a prospective case series conducted at the Department of Surgical Gastroenterology, Motilal Nehru Medical College, Prayagraj, between April 2024 and April 2026.

Inclusion criteria

All diagnosed cases of NCPH who presented to the outpatient department in the above time period and consenting to participate in the study.

Exclusion criteria

Patients with underlying cirrhosis, splenic vein thrombosis, uncorrectable coagulopathy, previous shunt surgery or splenectomy and those not giving consent for participation were excluded.

Approval from the institutional ethics committee was obtained.

Pre-operative workup

All patients underwent thorough history taking and clinical examination, routine blood investigations, upper gastrointestinal (GI) endoscopy (UGIE), and imaging (ultrasonography of the abdomen and triple phase CT scan of the abdomen). 

Patients with anaemia and thrombocytopenia were transfused with appropriate products preoperatively to achieve a hemoglobin of 10 g/dl and platelet count of 100,000 cu/mm before surgery. Informed consent for the surgery was taken from the patients. All patients received preoperative immunization with pneumococcal, meningococcal and *Hemophilus influenzae* type b vaccines, two weeks prior to the surgery. The patients were kept nil by mouth the night before surgery. A single dose of antibiotic (second-generation cephalosporin) was given one hour before the incision.

Operative technique

The surgery was performed under general anaesthesia with the patient placed in the supine position. A reverse Makucchi incision (L-shaped incision) was given (Figure 1) and abdomen was opened in layers. The liver was examined grossly for any evidence of cirrhosis (no biopsy was done). The spleen was mobilized from lateral to medial, after dividing the attachments and adhesions. After ligating and dividing the splenic artery, the splenic vein was clamped close to the splenic hilum, divided with scissors between the clamps and spleen was removed (Figure 2). Retroperitoneal dissection was then performed and left renal hilum was exposed. Left renal vein was identified as the anterior most structure in the hilum and was dissected further. The clamped splenic vein was further dissected after ligating small pancreatic tributaries and prepared for anastomosis. An end-to-side shunt was made between the splenic vein and left renal vein using 6-0 Prolene (Ethicon, Rariton, NJ, USA) (Figure 3) using the double needle technique. The diameter of the shunt was measured using a sterile measuring scale. The patency of the shunt was confirmed using handheld Doppler device. An abdominal drain (28 No.) was placed in the splenic fossa. Unfractionated heparin (UFH) was started intraoperatively as infusion at the rate of 16.5 U/kg/h and continued in the postoperative period (POD) for one day. This was followed by low-molecular-weight heparin (LMWH) at the dose of 0.6 mg once a day subcutaneously, starting from day two and transitioning to oral anticoagulant nicoumalone (dose 2 mg OD) by day five. The target International Normalized Ratio (INR) was kept between 2 and 3. Hematological parameters were checked on POD 1 and on POD 3. The abdominal drain was removed when the output was minimal for a day and subsequently the patients were discharged. Postoperatively, they were followed up in the outpatient department where suture/staple removal was done. Repeat Doppler ultrasonography (USG) was done at three months to confirm shunt patency, along with endoscopy to see for the regression of varices. INR was checked every month during follow-up and the dose of nicoumalone was titrated accordingly.

**Figure 1:**
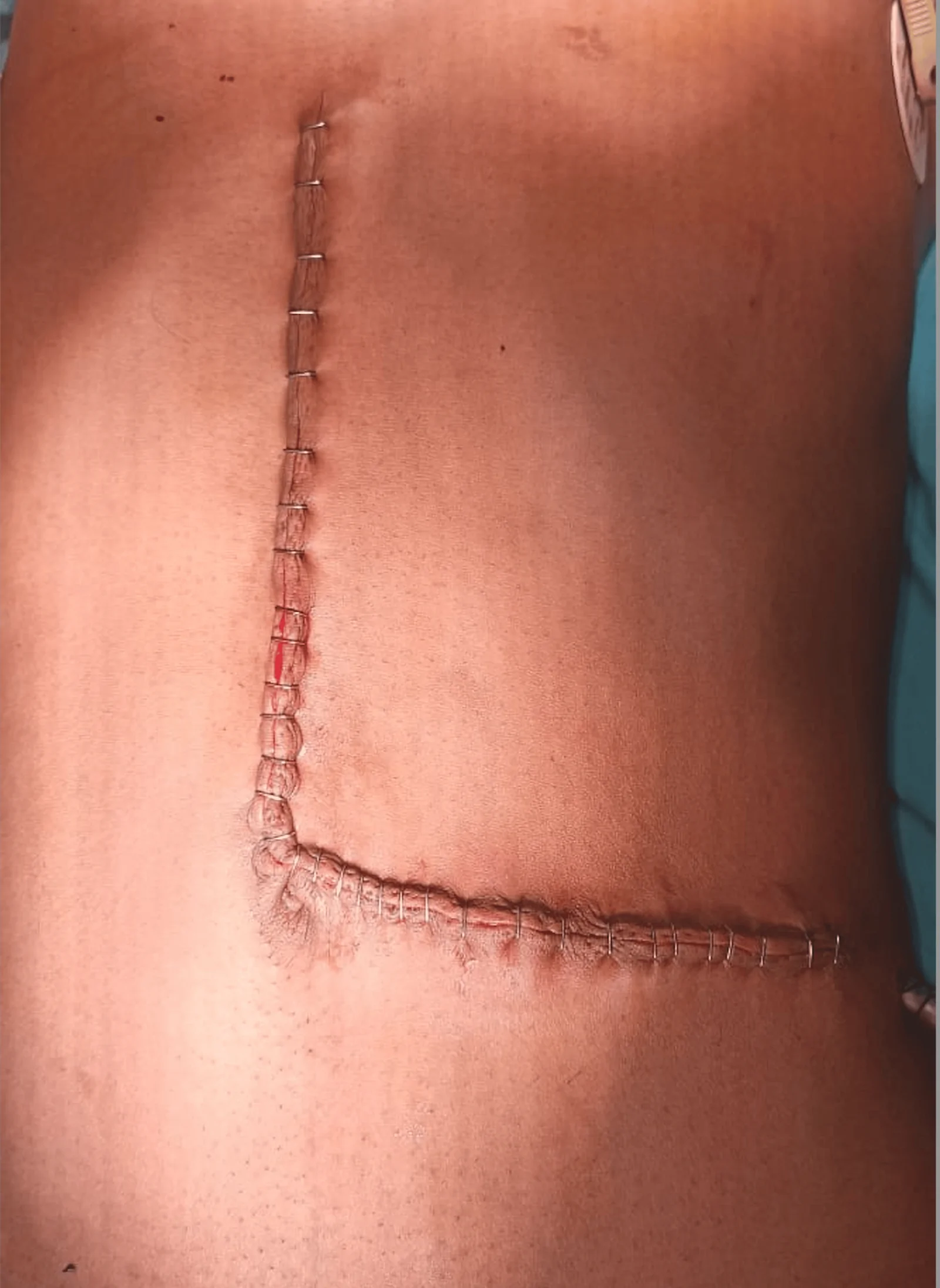
Reverse Makucchi incision

**Figure 2:**
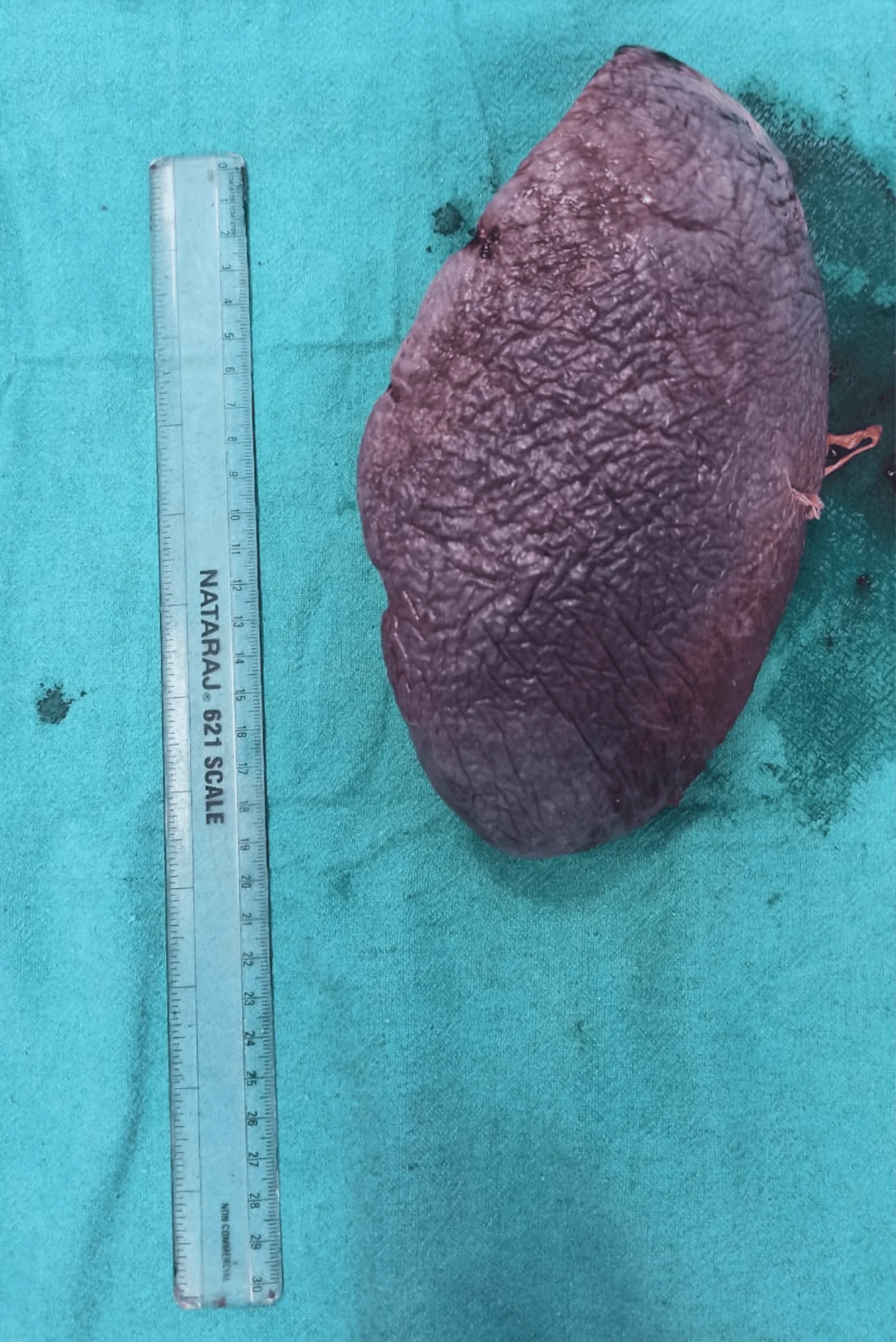
Resected spleen

**Figure 3:**
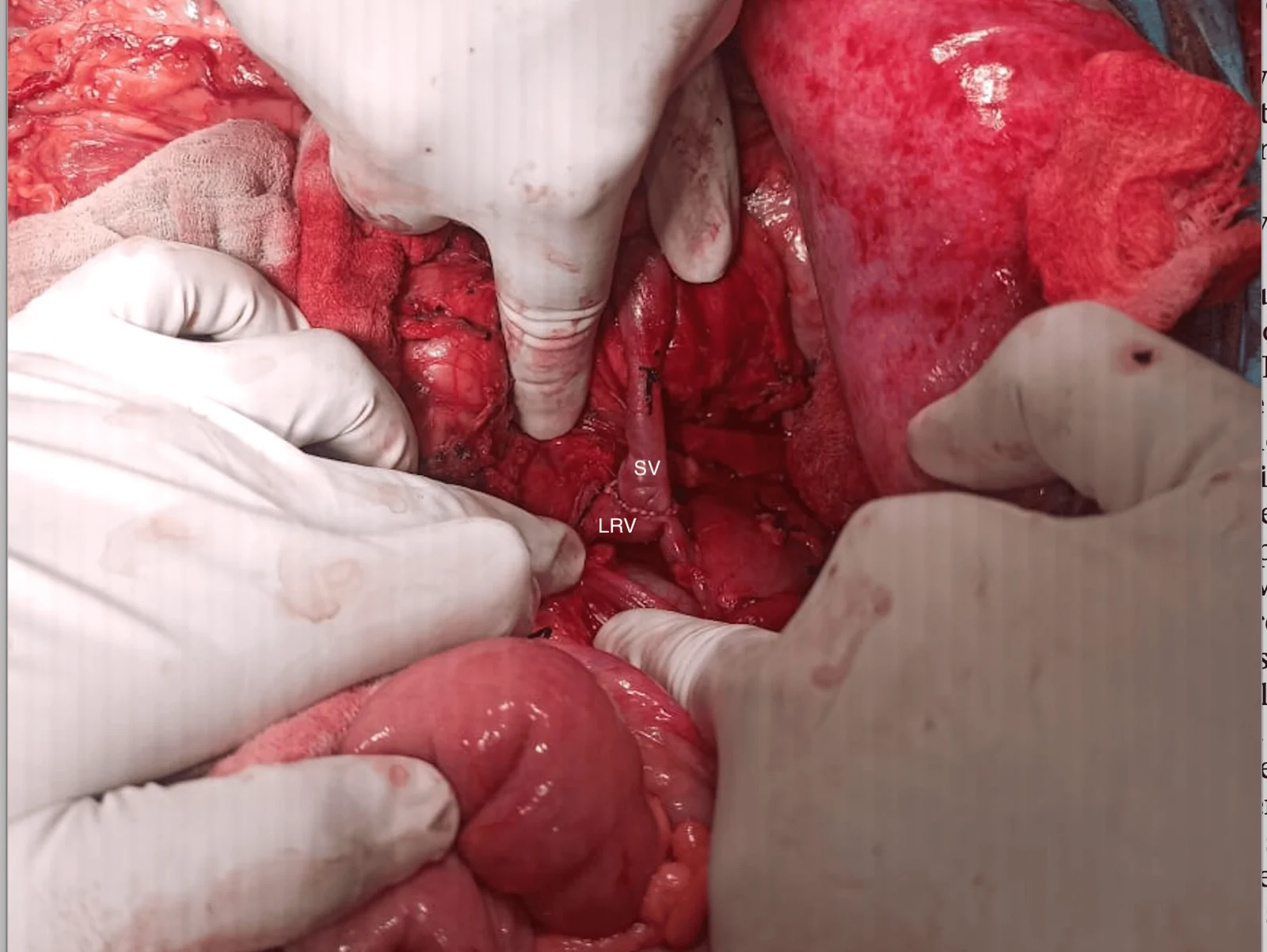
Completed end to side splenorenal anastomosis SV: Splenic vein; LRV: left renal vein.

## Results

Of the 15 patients who presented in this period, five were excluded due to the presence of cirrhosis and underwent splenectomy only. The remaining 10 patients were included in this series. A brief clinical profile of all the 10 patients is presented as follows.

Case 1

A 22-year-old male patient, presented with a history of lump in left upper abdomen and recurrent hematemesis since birth. On examination, he was found to have grade V splenomegaly. Hematology showed leucopenia and thrombocytopenia, while his liver function tests were normal. USG abdomen showed portal cavernomatous cholangiopathy (Figure 4) and a provisional diagnosis of EHPVO was made. A recent upper GI endoscopy showed grade IV esophageal varices with red colour signs, suggestive of recent bleed and endoscopic variceal ligation (EVL) was done. Subsequently, the patient was referred to us for PSRS. This patient was optimized by platelet transfusion preoperatively and triple vaccination was done as per the protocol. He underwent PSRS with an intraoperative blood loss of around 400 ml. His postoperative course was uneventful and the abdominal drain was removed on POD 5 and patient was discharged on POD 7. On follow-up Doppler ultrasound, which was done after three months, the shunt was found to be patent.

**Figure 4:**
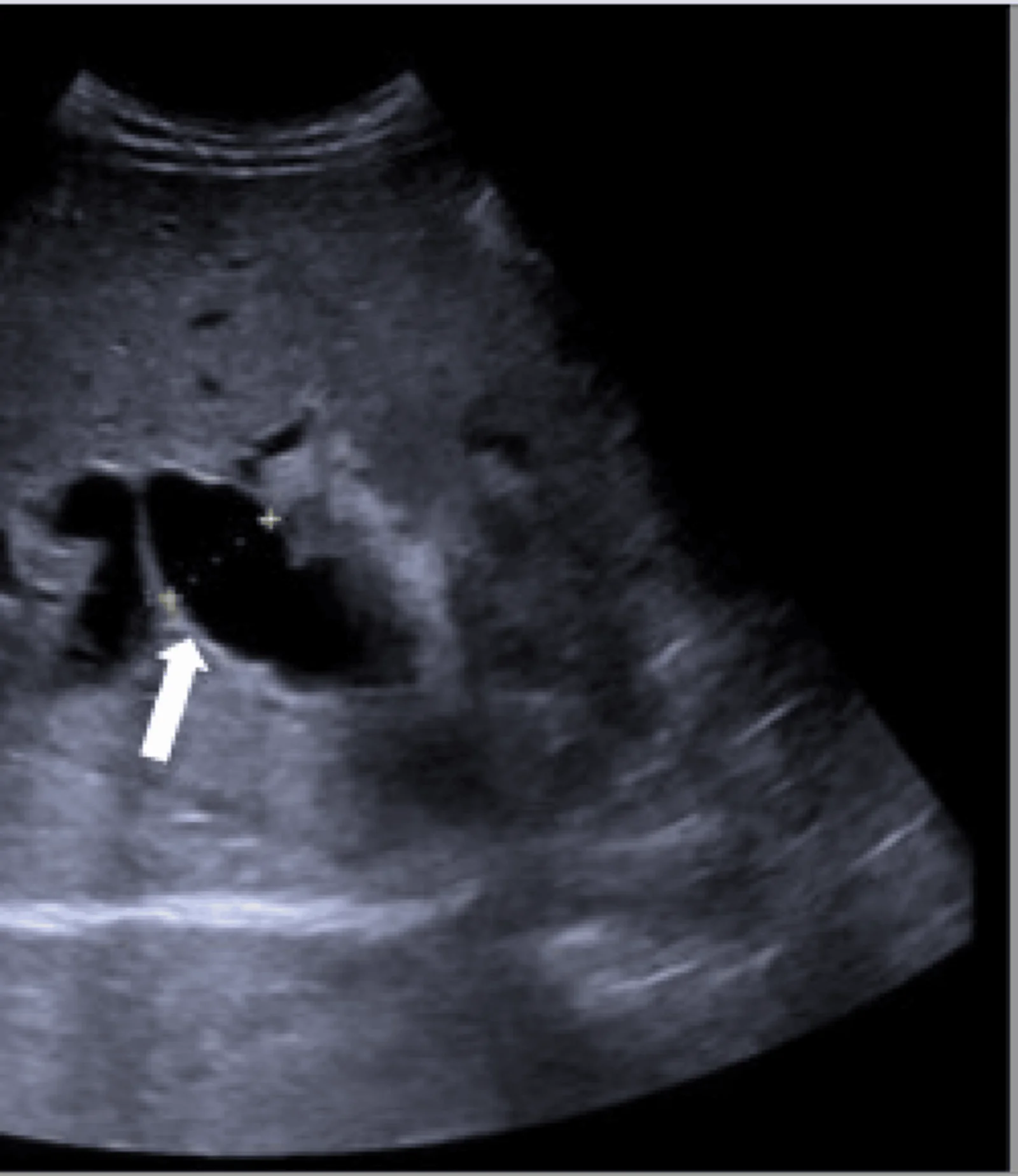
Ultrasonography showing portal cavernoma (white arrow)

Case 2

An 11-year-old boy presented with the history of poor growth and left upper abdominal lump for the last two years. On examination, he had a large spleen (grade III). Hematology and liver biochemistry were normal. USG abdomen showed portal cavernomatous changes, dilated splenic vein (diameter 14 mm) (Figure 5) and his endoscopy was unremarkable. A provisional diagnosis of EHPVO was made and he received triple vaccination two weeks prior to the surgery. He underwent PSRS (indication being moderate splenomegaly and growth retardation) and there was around 600 ml blood lost during the surgery. On POD 2, he complained of severe abdominal pain and distension. This was accompanied by a frank blood of >500 ml in the abdominal drain with tachycardia of 140/min. He was resuscitated with blood products and underwent re-exploration. There was no active bleeder identified, with multiple clots in the abdominal wall. Subsequently, he made an uneventful recovery and his drain was removed on POD 8 and he was discharged on POD 10.

**Figure 5:**
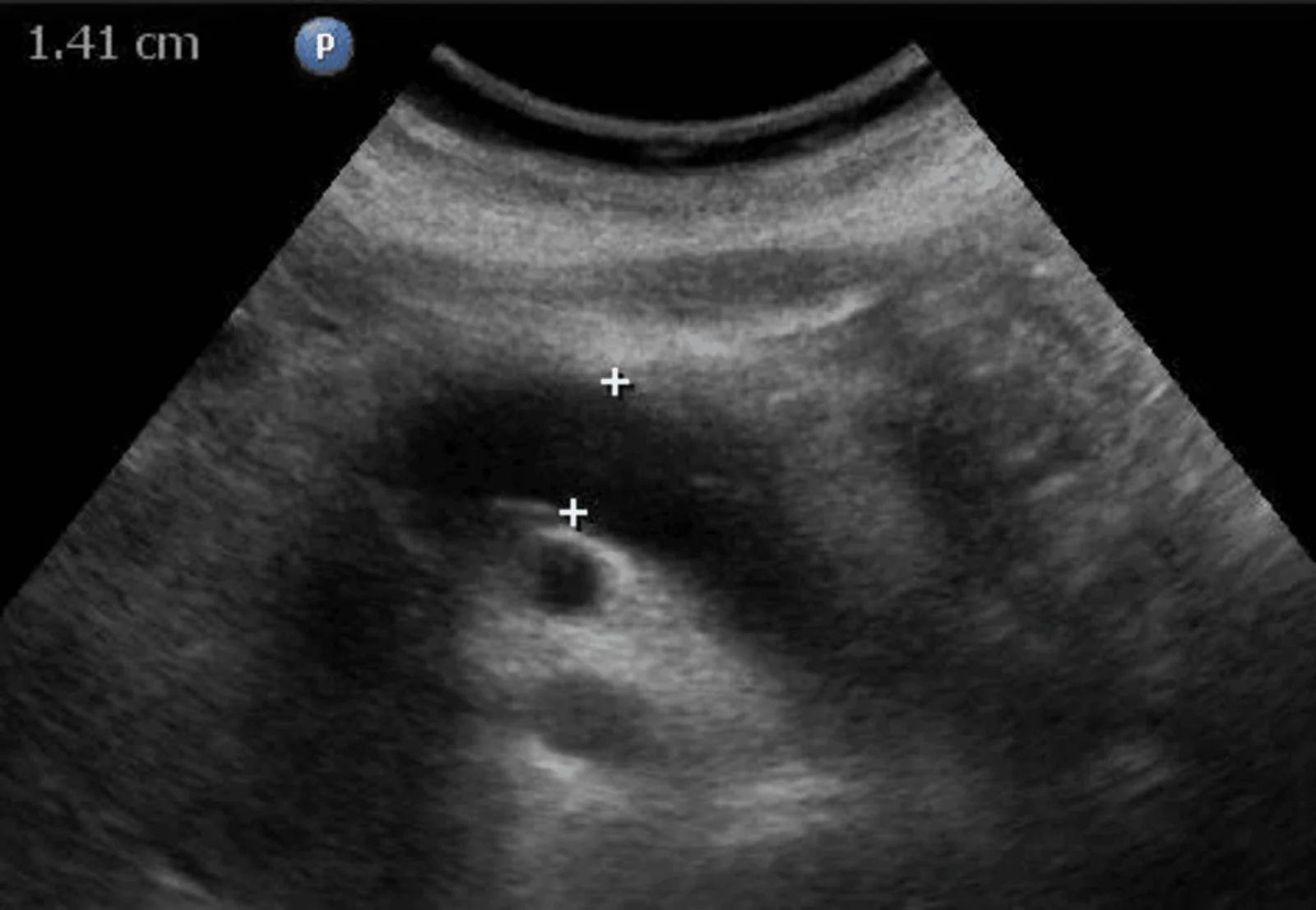
Ultrasonography showing dilated splenic vein (diameter 14 mm)

Case 3

A 25-year-old woman presented with menorrhagia, hematemesis and an abdominal lump for six months. On examination, she had massive splenomegaly. Hematology showed pancytopenia (hemoglobin of 4.7 g/dl, total leucocyte count of 1700 and a platelet count of 65,000). On abdominal sonography (Figure 6), there was dilated portal vein (diameter 18 mm). The left renal vein was retroaortic in course. A provisional diagnosis of NCPF was made and she was resuscitated with blood products. She underwent PSRS with a blood loss of 700 ml. On POD 1, she complained of dyspnea and right-sided chest pain. There were reduced breath sounds on right side and a chest X-ray revealed massive pleural effusion on the right side. An intercostal drain was inserted and it drained 750 ml of blood. Iatrogenic injury during central line insertion in the right internal jugular vein was attributed to be its cause. She improved gradually and was discharged on POD 15 after removal of the intercostal drain.

**Figure 6:**
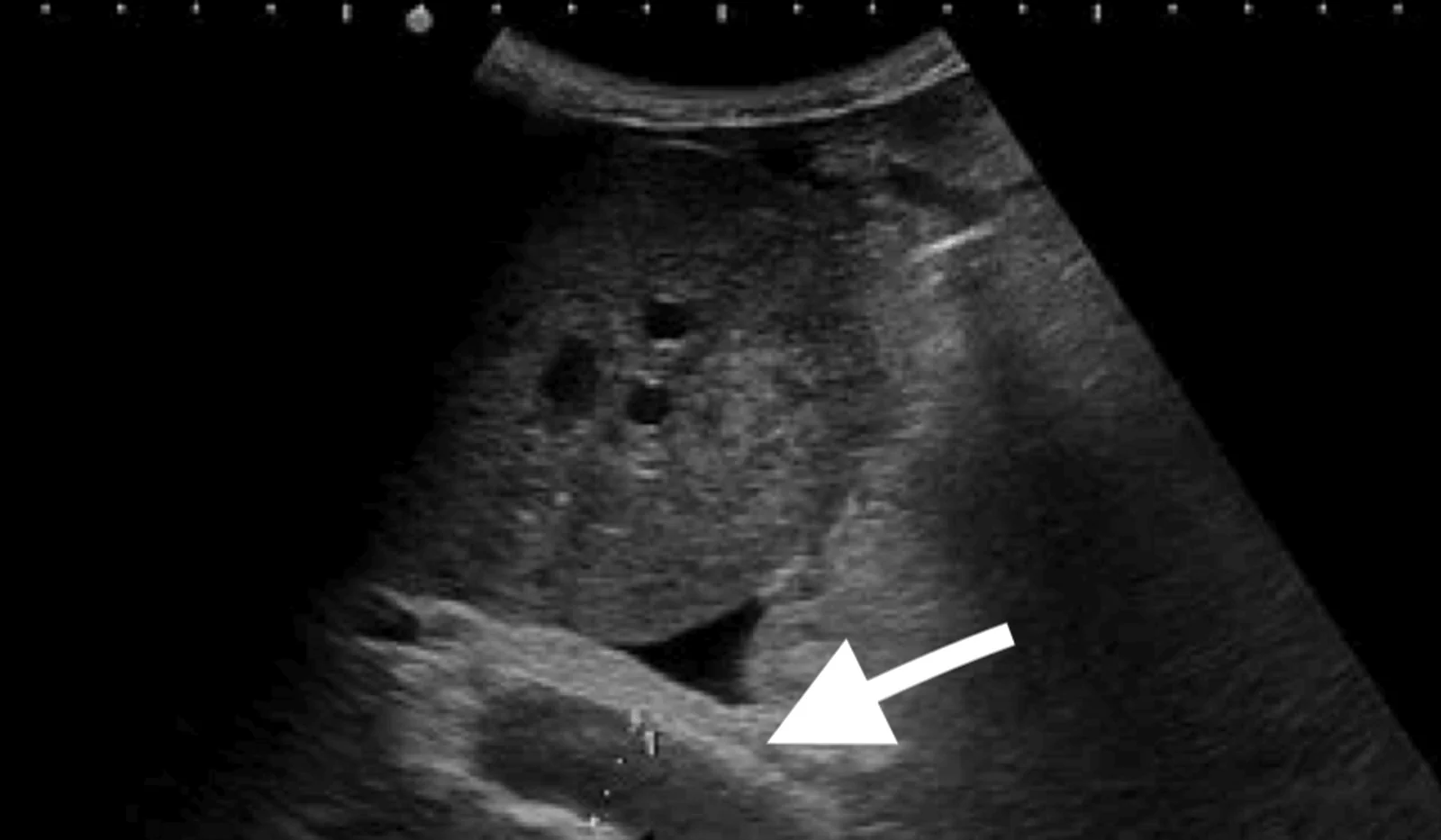
Ultrasonography showing dilated main portal vein (white arrow)

Case 4

A 17-year-old male patient presented with a history of abdominal lump and pain for four years. On examination, he was found to have grade II splenomegaly. Routine blood workup was normal. USG of the abdomen showed portal cavernoma (Figure 7a, 7b) and a provisional diagnosis of EHPVO was made. He had low-grade I esophageal varices on endoscopy. A decision was made to perform PSRS prophylactically. He underwent an uneventful surgery and was discharged on POD 7. Follow-up Doppler after three months demonstrated a patent shunt.

**Figure 7:**
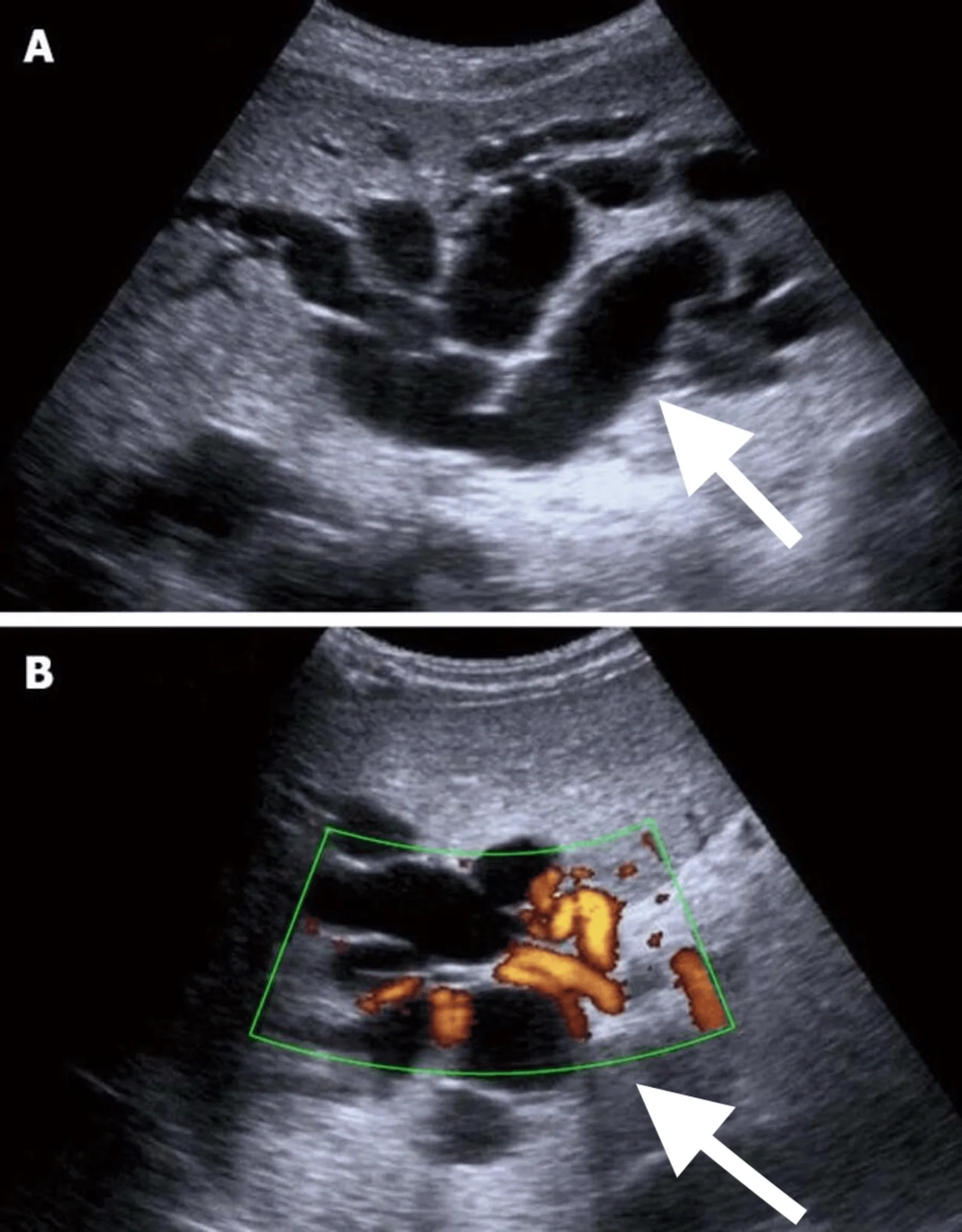
Ultrasonography showing (a) portal cavernoma (white arrow) and (b) colour flow image of cavernoma (white arrow)

Case 5

A 36-year-old woman presented with a history of an abdominal lump and epistaxis for four months. On examination, she was found to have grade II splenomegaly. She had a low platelet count of 50,000. Portal vein diameter of 13.4 mm was noted on USG (Figure 8). A provisional diagnosis of NCPF was made based on imaging findings and she underwent PSRS. Her postoperative course was uneventful and she was discharged on POD 8.

**Figure 8:**
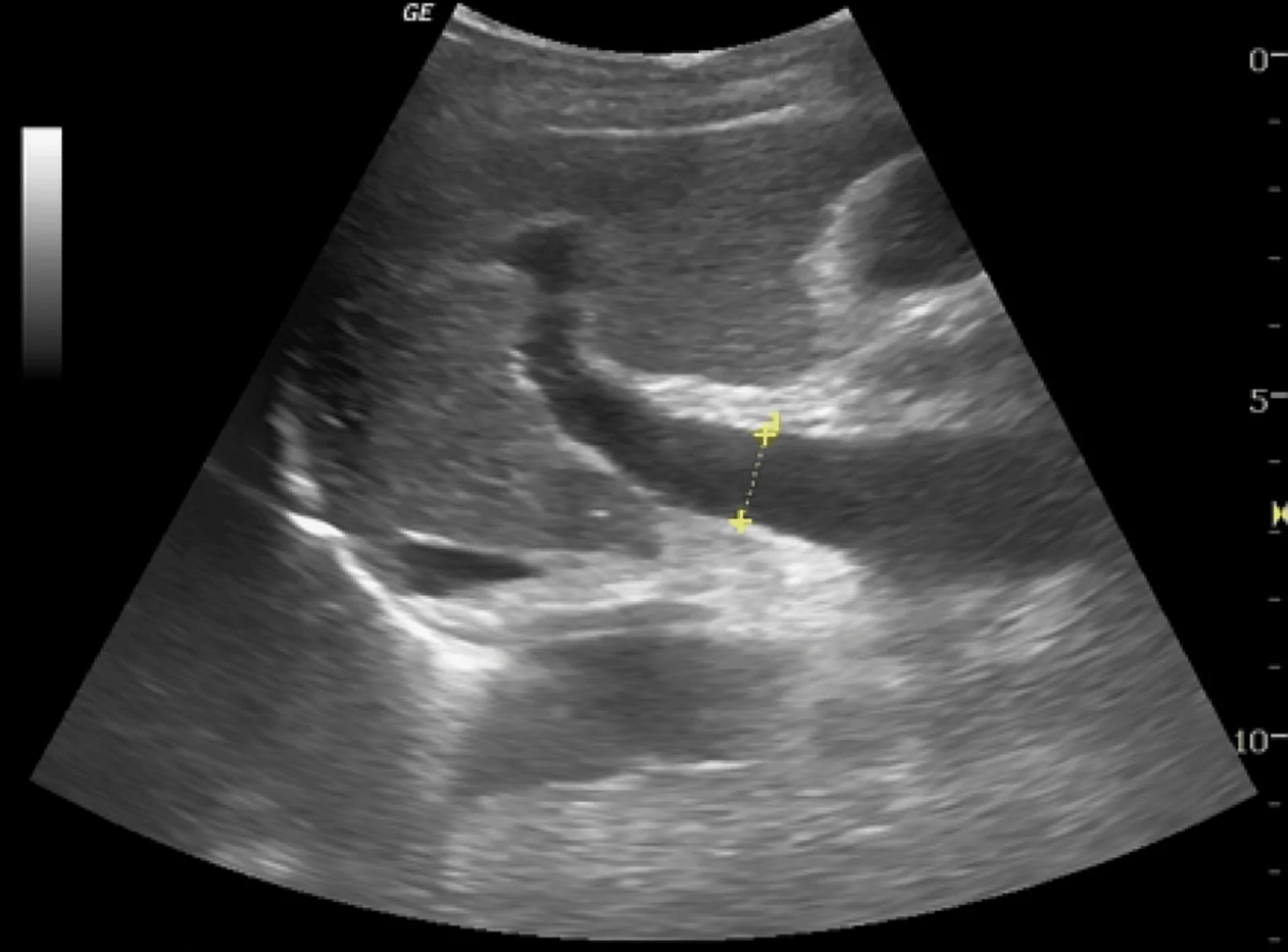
Ultrasonography showing portal vein of size 13.4 mm

Case 6

A 24-year-old lady presented with a history of recurrent hematemesis and progressive left-sided abdominal lump for the past 10 years. She was found to have moderate splenomegaly and had pancytopenia on routine blood workup. USG abdomen was suggestive of portal cavernoma (Figure 9) and a provisional diagnosis of EHPVO was made. She underwent PSRS after adequate optimization. On POD 1, she had 400 ml of blood in her abdominal drain. Oral anticoagulant was withheld and she was monitored. Subsequently, her drain output became serous and the drain was removed on POD 7. She was discharged on POD 9.

**Figure 9:**
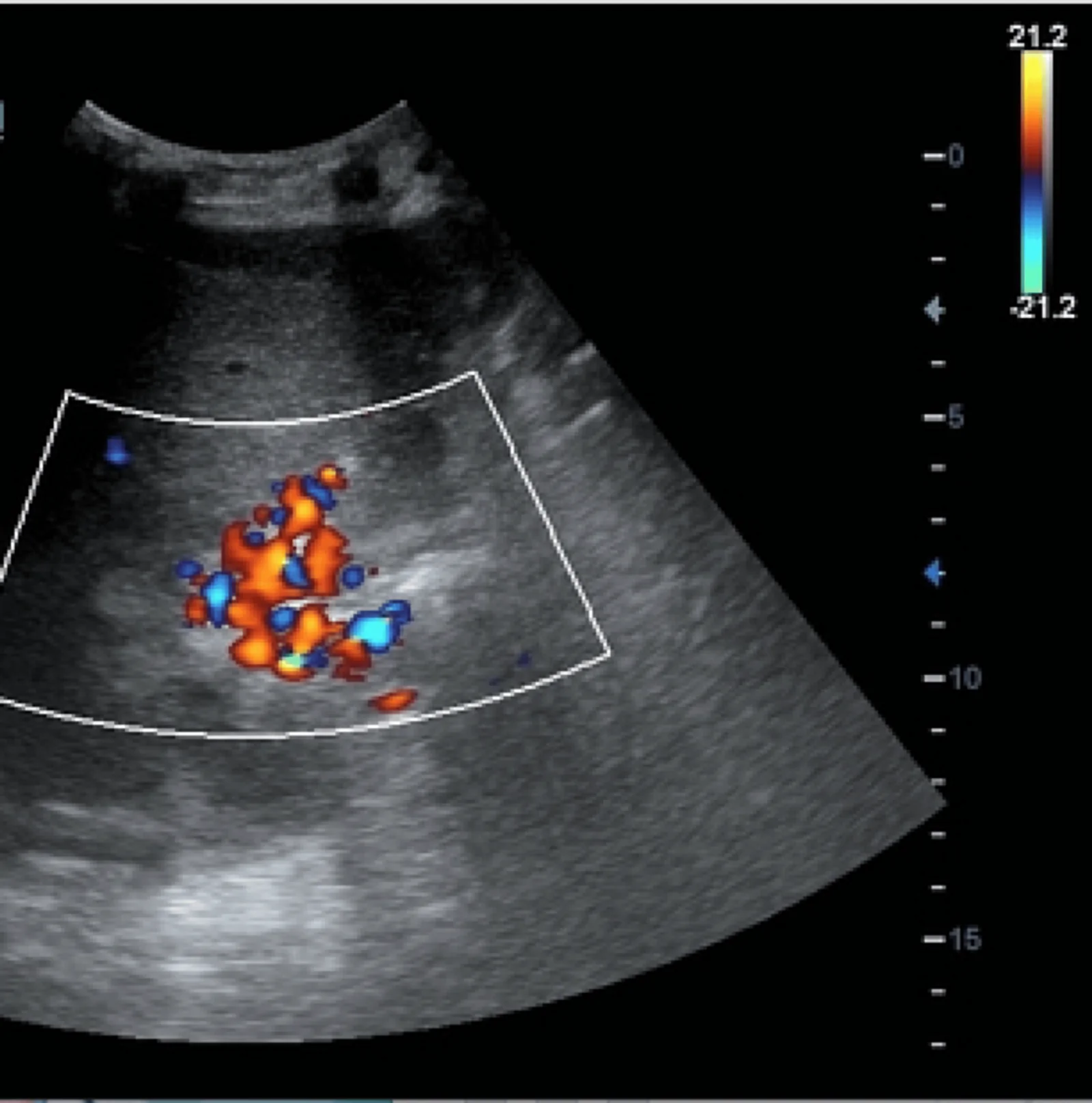
Ultrasonography showing portal cavernoma (colour flow image)

Case 7

An 18-year-old male patient presented with a history of a large abdominal lump, progressively increasing since childhood, along with two episodes of hematemesis for which endoscopic variceal ligation was done successfully. He had massive splenomegaly and pancytopenia. USG abdomen was suggestive of EHPVO (FIgure 10). He underwent PSRS to prevent recurrent bleeding and his postoperative recovery was uneventful.

**Figure 10:**
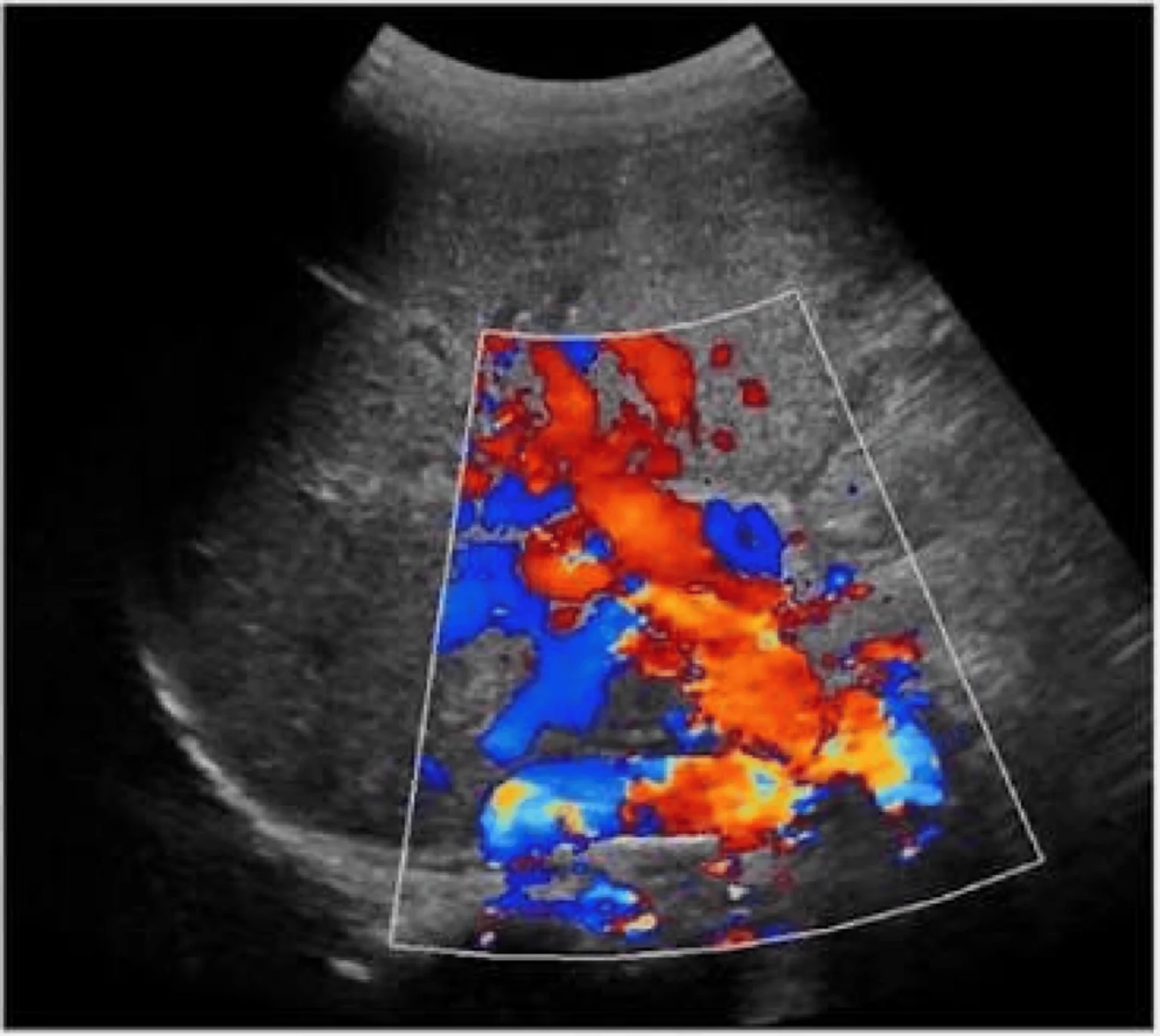
Ultrasonography showing colour flow image of extrahepatic portal vein obstruction (EHPVO)

Case 8

A 23-year-old male patient presented with a history of upper abdominal lump since six months. On examination, he was found to have splenomegaly. His blood panels revealed thrombocytopenia. USG abdomen showed a dilated main portal vein (19 mm diameter) and a diagnosis of NCPF was made (FIgure 11). He underwent PSRS. Intraoperatively, there were dense adhesions of the spleen to the abdominal wall and the left lobe of the liver, requiring extensive dissection. This patient had significant postoperative bleed on POD 2, and his drain output was 600 ml of blood. His anticoagulants were stopped and he was transfused packed red blood cells. Subsequently, his hemodynamics improved and he was discharged on POD 8.

**Figure 11:**
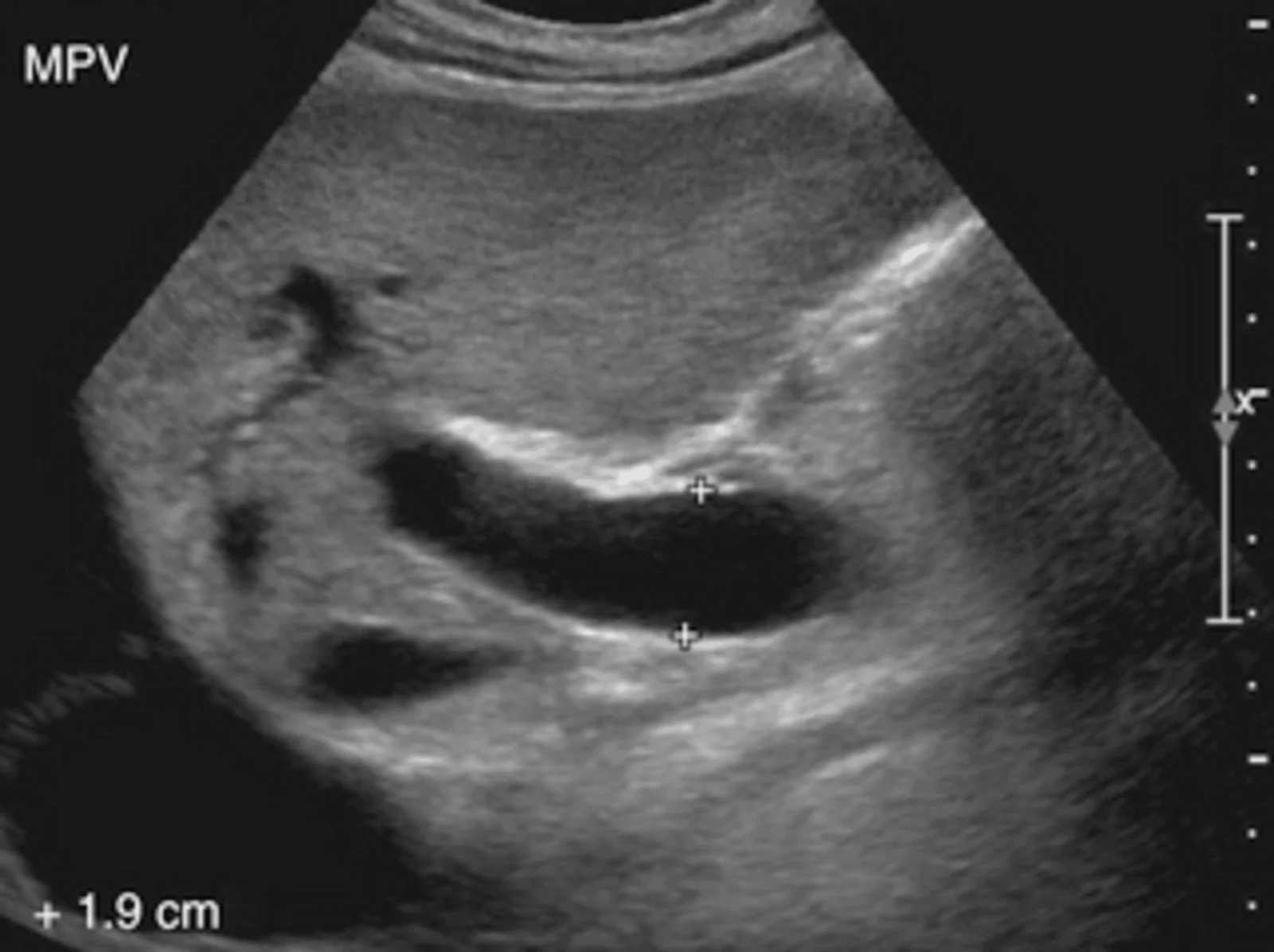
Ultrasonography showing dilated main portal vein (MPV) of 19 mm

Case 9

An 18-year-old male patient presented with complaints of dull aching pain on the left side of the abdomen, along with a vague lump for two years. He also gave a history of intermittent hematuria and recurrent cough. On examination, there was grade III splenomegaly and infiltrates on the chest X-ray. He had bicytopenia (TLC of 2500 and platelet count of 75,000). Upper GI endoscopy revealed grade II esophageal varices (Figure 12) for which EVL was done prophylactically. USG abdomen revealed a dilated main portal vein and a provisional diagnosis of NCPF was made. He was started on antibiotics and underwent PSRS subsequently. His intra- and postoperative course was uneventful.

**Figure 12:**
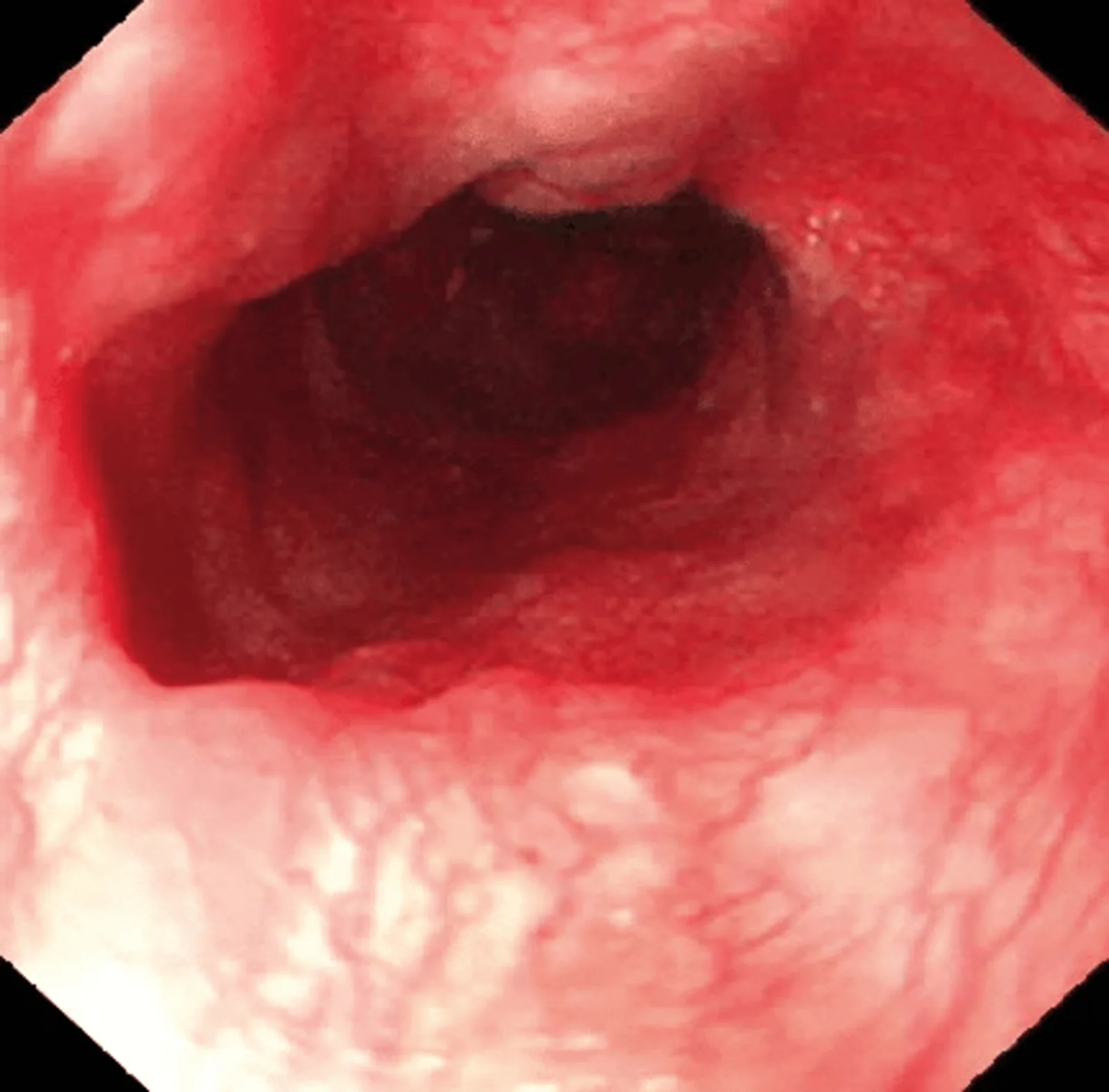
Endoscopy showing grade II esophageal varices

Case 10

A 32-year-old woman presented with a history of upper abdominal lump and menorrhagia since one year. Examination revealed grade II splenomegaly with pallor. Hematology showed pancytopenia. A provisional diagnosis of NCPF was made based on the dilated portal vein on abdominal sonography (Figure 13). She underwent PSRS and made an uneventful recovery. She was discharged on POD 7.

**Figure 13:**
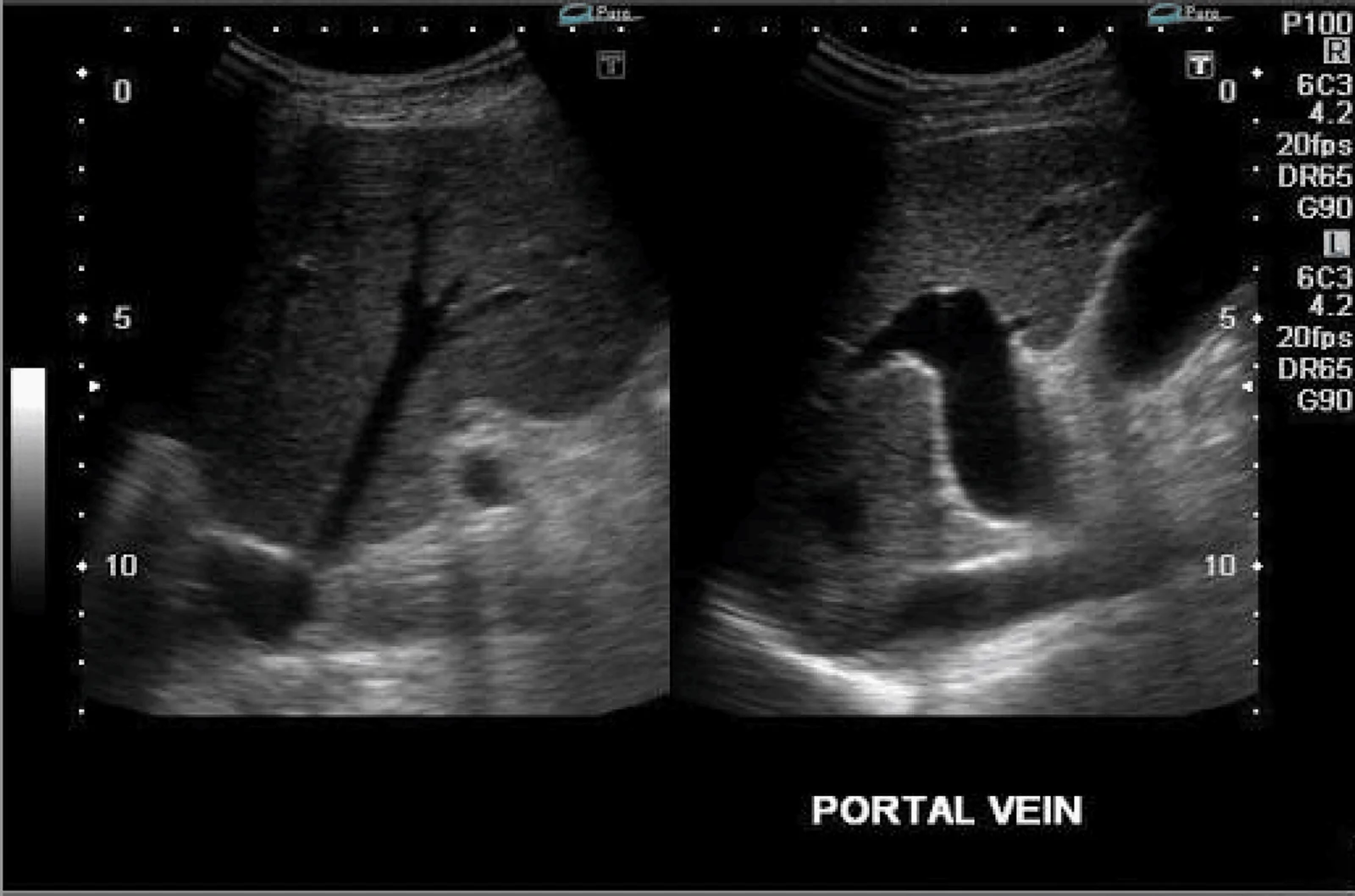
Ultrasonography showing dilated portal vein

The demographic and clinical features of these patients are summarized in Table 1. The radiological and endoscopic findings are summarized in Table 2. The intraoperative findings and postoperative course are summarized in Table 3.

**Table 1:** Demographic and clinical features of the 10 patients M: Male, F: Female, Y: Yes, N: No, LUQ: left upper quadrant, EVL: endoscopic variceal ligation, WNL: within normal limits.

Parameter	Case 1	Case 2	Case 3	Case 4	Case 5	Case 6	Case 7	Case 8	Case 9	Case 10
Age/Sex	22/M	11/M	25/F	17/M	36/F	24/F	18/M	23/M	18/M	32/F
Duration of symptoms (in years)	22	2	1/2	4	1/3	10	15	1/2	2	1
LUQ lump	Y	Y	Y	Y	Y	Y	Y	Y	Y	Y
LUQ pain	N	N	N	Y	Y	Y	Y	N	Y	N
Hematemesis	Y	N	Y	N	N	Y	Y	N	N	N
Other bleeding	N	N	Y	N	Y	N	N	N	Y	Y
Growth retardation	N	Y	N	N	N	N	Y	N	N	N
Ascites	N	N	N	N	N	N	N	N	N	N
Splenomegaly (grade)	Present: Grade 5	Present: Grade 3	Present: Grade 5	Present: Grade 2	Present: Grade 2	Present: Grade 3	Present: Grade 5	Present: Grade 2	Present: Grade 3	Present: Grade 2
History of EVL	Y	N	Y	N	N	Y	Y	N	N	N
Haemoglobin (Ref: 12.0-17.5 g/dl)	14.6	10.3	4.7	13.2	10.4	9.5	7.2	11.2	10.8	8.5
Total leucocyte count (Ref: 4000-11,000 cu.mm)	3400	4600	1700	6150	3500	3200	2300	4000	2500	3800
Platelet count (Ref: 150,000-450,000 cu.mm)	50,000	100,000	65,000	68,000	50,000	40,000	30,000	55,000	75,000	65,000
Liver function tests	WNL	WNL	WNL	WNL	WNL	WNL	WNL	WNL	WNL	WNL

**Table 2:** Radiological and endoscopic findings of the ten patients USG: Ultrasonography; EHPVO: extrahepatic portal vein obstruction; NCPF: non-cirrhotic portal fibrosis; IHBRD: Intrahepatic biliary radicle dilatation, LRV: left renal vein, UGIE: upper gastrointestinal endoscopy.

USG/CT findings										
Parameter	Case 1	Case 2	Case 3	Case 4	Case 5	Case 6	Case 7	Case 8	Case 9	Case 10
Cirrhosis	N	N	N	N	N	N	N	N	N	N
Portal vein diameter in mm (if seen)	N	N	18	N	13.4	N	N	19	17	16
Portal cavernoma	Y	Y	N	Y	N	Y	Y	N	N	N
IHBRD	Y	Y	N	Y	N	Y	Y	N	N	N
Ascites	N	N	N	N	N	N	N	N	N	N
Spleen size (in cm)	22.3	17.2	22	16.9	19	17	21.4	15.3	17.5	15.5
Splenic vein diameter (if seen)	Seen: 16 mm	Seen: 14 mm	Seen: 14 mm	Seen: 13 mm	Seen: 12 mm	Seen: 15 mm	Seen: 13 mm	Seen: 14 mm	Seen: 15 mm	Seen: 16 mm
Left renal vein diameter	12 mm	11 mm	13 mm	10 mm	10 mm	11 mm	12 mm	12 mm	10 mm	9 mm
Anatomical anomalies	N	N	Retroaortic LRV	N	N	N	N	N	N	N
Diagnosis	EHPVO	EHPVO	NCPF	EHPVO	NCPF	EHPVO	EHPVO	NCPF	NCPF	NCPF
UGIE findings										
Esophageal varices and their grade	Grade 4	N	Grade 2	Grade 1	N	Grade 3	Grade 4	N	Grade 2	Grade 1
EVL done	Y	N	Y	N	N	Y	Y	N	Y	N
Gastric varices	N	N	N	N	N	N	N	N	N	N
Ectopic varices	N	N	N	N	N	N	N	N	N	N
Signs of recent hemorrhage	Y	N	N	N	N	N	N	N	N	N

**Table 3:** Intraoperative findings and postoperative course of the ten patients Postoperative hemoglobin, total leucocyte count (TLC) and platelet counts mentioned in the table were done on POD 1.

Intraoperative findings										
Parameter	Case 1	Case 2	Case 3	Case 4	Case 5	Case 6	Case 7	Case 8	Case 9	Case 10
Liver	Normal	Normal	Normal	Normal	Normal	Normal	Normal	Normal	Normal	Normal
Ascites	N	N	N	N	N	N	N	N	N	N
Intraoperative blood loss (ml)	400	600	700	550	300	450	400	500	650	350
Duration of surgery (in min)	275	285	320	265	300	325	290	310	300	280
Intraoperative complication	N	N	N	N	N	N	N	N	N	N
Postoperative course										
Bleeding	N	Y	Y	N	N	Y	N	Y	N	N
Jaundice	N	N	N	N	N	N	N	N	N	N
Encephalopathy	N	N	N	N	N	N	N	N	N	N
Relaparotomy	N	Y	N	N	N	N	N	N	N	N
Hemoglobin (in g/dl)	12.8	8.4	10.6	11.5	12.4	11.3	10.4	11.0	10.5	9.6
TLC (in cu.mm)	7600	5000	7300	9500	11400	10700	8400	10900	8700	9900
Platelet count (in cu.mm)	113,000	125,000	118,000	137,000	165,000	108,000	121,000	151,000	141,000	154,000
Drain removal (in days)	5	8	7	4	5	7	5	6	4	5
Duration of hospital stay (in days)	7	10	15	7	8	9	7	8	6	7

## Discussion

Portal hypertension is a clinical syndrome defined by a hepatic venous pressure gradient (HVPG) >5 mmHg. However, HVPG measurement is unsuitable to diagnose NCPH because the liver architecture is preserved [1]. NCPH constitutes up to 30% of patients with portal hypertension [1,3]. NCPH affects individuals in their childhood or in the prime of their lives, that is, 10 to 30 years of age [4]. It mainly includes NCPF (20%-30% cases of portal hypertension) and EHPVO (5%-10% cases of portal hypertension) [4].

NCPF is characterized by a lack of a globally accepted definition and a varied geographical distribution. The incidence has decreased in India due to better hygiene, access to safe water and improved perinatal care [5]. It has a slight male preponderance in India (M:F is 2:1), whereas it is more common in females in Japan (M:F is 1:3) [6]. In our series, five patients had NCPF, of which three were women (M:F is 1:1.5). Three patients with NCPF belonged to the age group of 25-35 years, while two patients were younger than 25 years (mean was 26.8±6.43 years). This matches the age range of the patients with NCPF, which is between 25 and 35 years [1].

EHPVO is defined as “a vascular disorder of the liver, characterized by obstruction of the extra-hepatic portal vein with or without involvement of intrahepatic portal vein radicles, splenic vein, or superior mesenteric vein” [7]. A portal cavernoma or a recent thrombus was included in the definition after the Baveno V consensus [8]. In our study, out of five patients with EHPVO, four were men. This is similar to other studies that also report that it is more common in men (M:F is around 1.56:1) [9].

EHPVO can start as early as six weeks after birth and has a bimodal distribution of presentation (at around three years and at eight years of age) in childhood. The mean age of presentation is 6.3 years in childhood [4]. However, the median age in adults is 16.5 years (interquartile range (IQR): 6.7-30 years), as per a study by Pati et al. [9]. In our study, the mean age was 18.4±4.49 years. This value is higher than other high-volume centers where a robust pediatric gastroenterology department is able to pick these patients at a younger age and refer them for early surgery.

The etiopathogenesis involves obliterative portal venopathy due to recurrent abdominal infections in pediatric age or due to exposure to heavy metals like arsenic or vinyl chloride in genetically predisposed individuals. These changes occur in the main portal vein in EHPVO, whereas it occurs in intrahepatic, presinusoidal portal vein branches in NCPF [1].

In our series, all patients reported a lump arising from the left upper quadrant and extending towards the umbilicus. Three of them had the lump crossing the umbilicus (massive splenomegaly, grade V and a mean size of 21.9±0.37 cm). Overall, the mean splenic size was 18.4±2.41 cm. In a study by Khanna and Sarin [4], the mean size of the spleen was around 11 cm below the costal margin (normal spleen is around 9-11 cm in size). In our study, 40% of the patients reported hematemesis, while two patients with NCPF also had menorrhagia and one of them had epistaxis. One of the NCPF patients also complained of hematochezia. This corresponds to the Khanna and Sarin's study [10] where variceal bleeding was present in 50%-85% patients. Growth retardation was present in 40% of patients of EHPVO, similar to the study by Mir et al. [11]. Since our series has a smaller number of patients, it is difficult to draw any significant conclusions from this finding.

All patients had thrombocytopenia, 70% had leucopenia and 30% had pancytopenia. In a study by Rajalingam et al. [12], symptomatic hypersplenism was reported in 70.3% of patients with EHPVO, while Khanna and Sarin [4] reported hypersplenism in 65%-84% of patients with NCPF. All patients had normal liver function tests, suggesting preserved liver architecture. On imaging, all patients with EHPVO had asymptomatic portal biliopathy and intrahepatic biliary radicle dilatation (IHBRD), similar to the findings in the study by Dhiman et al. [3] (portal biliopathy in 90% of patients, of which 80% are asymptomatic). None of them had chronic liver disease on imaging or ascites. The mean diameter of splenic vein (SV) was 14.2±1.24 mm and that of left renal vein (LRV) was 11±1.18 mm. One patient with NCPF had retroaortic LRV, which was identified on cross-sectional imaging. Variations in the anatomy of LRV exist in 0.1%-10% of the cases across various studies [13]. Of these, the commonest is circumaortic LRV (venous collar) in about 4%-7% of the cases, followed by retroaortic LRV in 1%-3% of the cases.

Oesophagogastroduodenoscopy (OGD) showed esophageal varices in 70% of the patients. The prevalence of esophageal varices varies between 60% and 90% across various studies on NCPF and EHPVO [14]. There were no signs of recent hemorrhage or gastric or ectopic varices in any of these patients except in one patient with grade IV esophageal varices.

Intraoperatively, there was no ascites, and the liver was grossly normal in all patients. All patients underwent splenectomy with proximal splenorenal shunt. The mean amount of blood loss during the surgery was 490±126.09 ml, and the mean duration of surgery was 295±18.57 minutes. Anand et al. [15] reported a mean blood loss of 865 ml and almost similar duration of surgery (270 minutes) in their study on the role of PSRS in gastric varices in NCPH.

Postoperatively, none of the patients developed jaundice or encephalopathy. The incidence of post-shunt encephalopathy is around 12%-13% in different studies [16,17]. It is less in NCPH than in cirrhotic patients due to the preserved liver function. It occurs due to gut toxins like ammonia entering into systemic circulation, bypassing the liver and manifesting as personality changes, confusion, and stupor. It is managed by increasing the intake of dairy protein, oral lactulose, rifaximin and shunt occlusion in severe cases, with a contradictory risk of bleeding.

The incidence of shunt thrombosis is 7%-11% [18]. The shunt patency rate is around 43% at the end of five years in some studies [19]. In the immediate postoperative period, technical factors like the technique of anastomosis, type of suture material, lie of shunt, flow and size of vein, presence of plaques in the venous wall affect patency. In the delayed phase, underlying procoagulant disorder, post-splenectomy thrombocytosis and decreased flow due to splenectomy are important. Mishra et al. [19] examined the factors affecting long-term shunt patency in 50 patients undergoing PSRS over 10 years and found splenic vein diameter as the only significant factor. Patency is higher in NCPF than in EHPVO, but it was not statistically significant in this study. Doppler ultrasonography underestimates shunt patency and dynamic CT portography is the preferred noninvasive investigation for detecting shunt patency, as it has an excellent correlation with conventional angiography, which is the gold standard for detecting shunt patency [19]. Rebleeding occurs in 10%-20% of the cases [12] and can be managed endoscopically. Anticoagulation helps in cases with an underlying hypercoagulable state.

Overall, the incidence of rebleeding after PSRS is 2.5%-10%, which is significantly lower than endoscopic variceal ligation or sclerotherapy (around 10%-20%) [12]. In our study, postoperative rebleeding occurred in four out of 10 cases (40%), but none was related to shunt thrombosis and none of them manifested as hematemesis or malena. Two of the patients with EHPVO had bleeding manifested through the drain, with persistent tachycardia. One of them was managed conservatively, while the other required re-exploration on POD 2. Overdose of anticoagulant in the perioperative period was believed to be the probable cause of bleeding in these two cases. Two patients with NCPF also developed bleeding postoperatively, one of which is iatrogenic and due to central line insertion.

The mean duration of abdominal drain removal was 5.6±1.28 days, and the mean duration of hospitalization was 8.4±2.45 days. This was comparable to the study by Anand et al. [15].

PSRS effectively decompressed the portal system and corrected hypersplenism in all cases (Table 3). Anticoagulation was discontinued at one year post-surgery, after follow-up imaging confirmed shunt patency. The need for long-term anticoagulation is debatable. Most of the evidence comes from retrospective studies, and short-term anticoagulation for three to six months is recommended to prevent shunt thrombosis [20]. In patients with underlying thrombophilic disorder, anticoagulants are given for an indefinite period or lifetime [20]. The challenge is to balance the risk of variceal rebleeding. There is emerging evidence of combining anticoagulation with antithrombotic therapy to counter postsplenectomy thrombocytosis, which contributes to shunt blockage [20].

Our short-term outcomes reaffirm PSRS as a safe, one-time, and effective procedure in NCPH, avoiding the need for repeated endoscopic intervention for bleeding and reducing the need for frequent blood transfusions. The limitations include a small group of patients, lack of liver biopsy for confirmed diagnosis of NCPF and short follow-up (the last two patients had a follow-up of less than three months at the time of writing this manuscript).

## Conclusions

PSRS is a reliable surgical option for patients with NCPH presenting with hypersplenism or recurrent variceal bleeding. The procedure offers excellent decompression, minimal mortality, and manageable morbidity. A teamwork by endoscopists, radiologists, and surgeons allows a tailored approach to manage these patients who have a protean and complex clinical profile.
